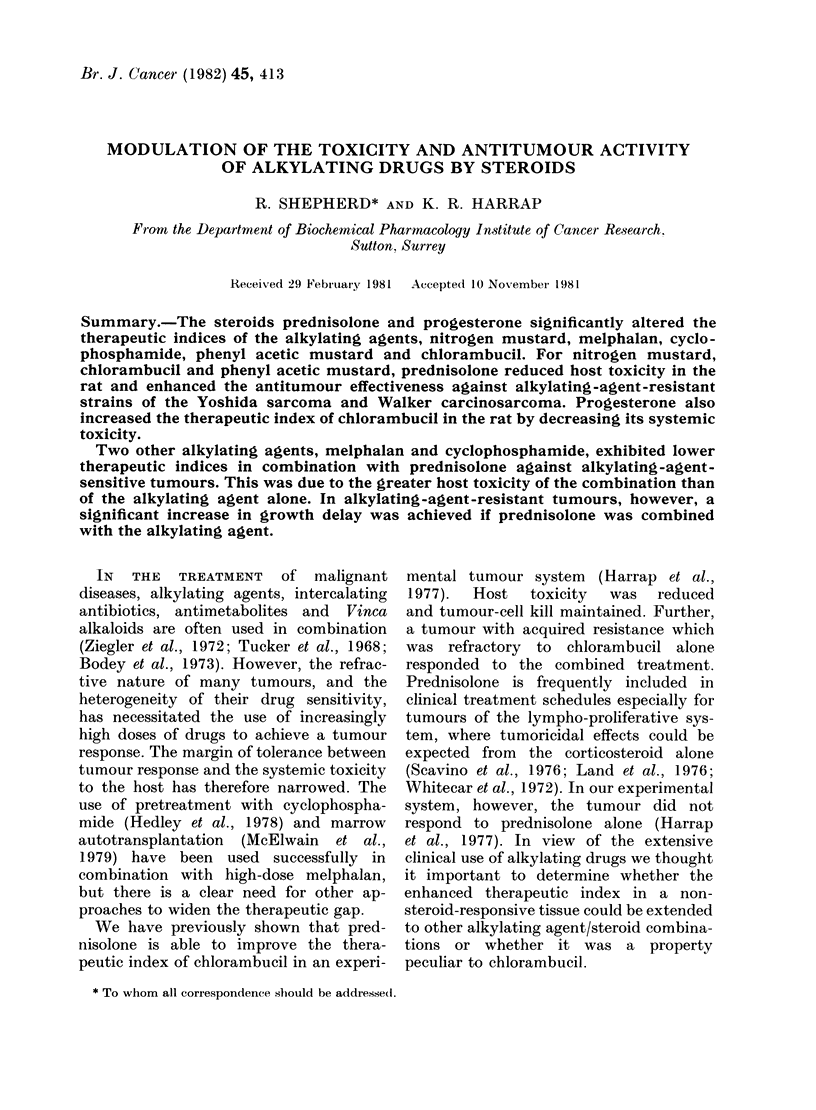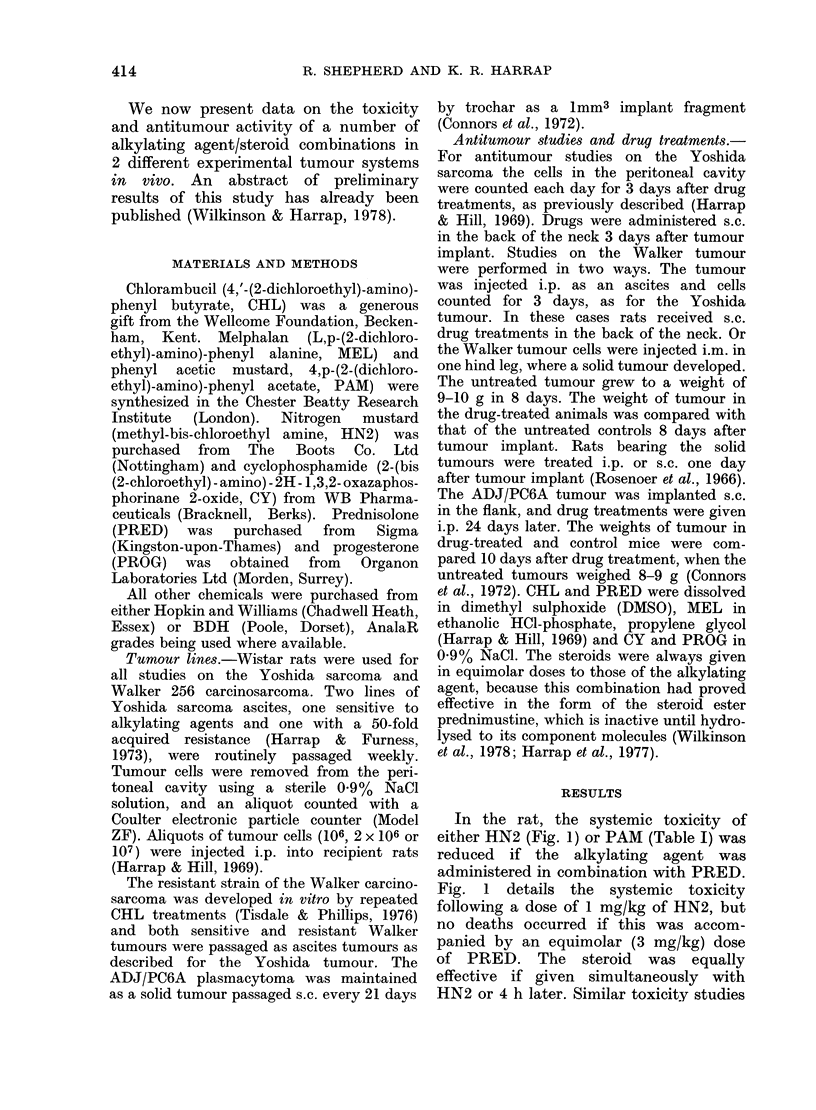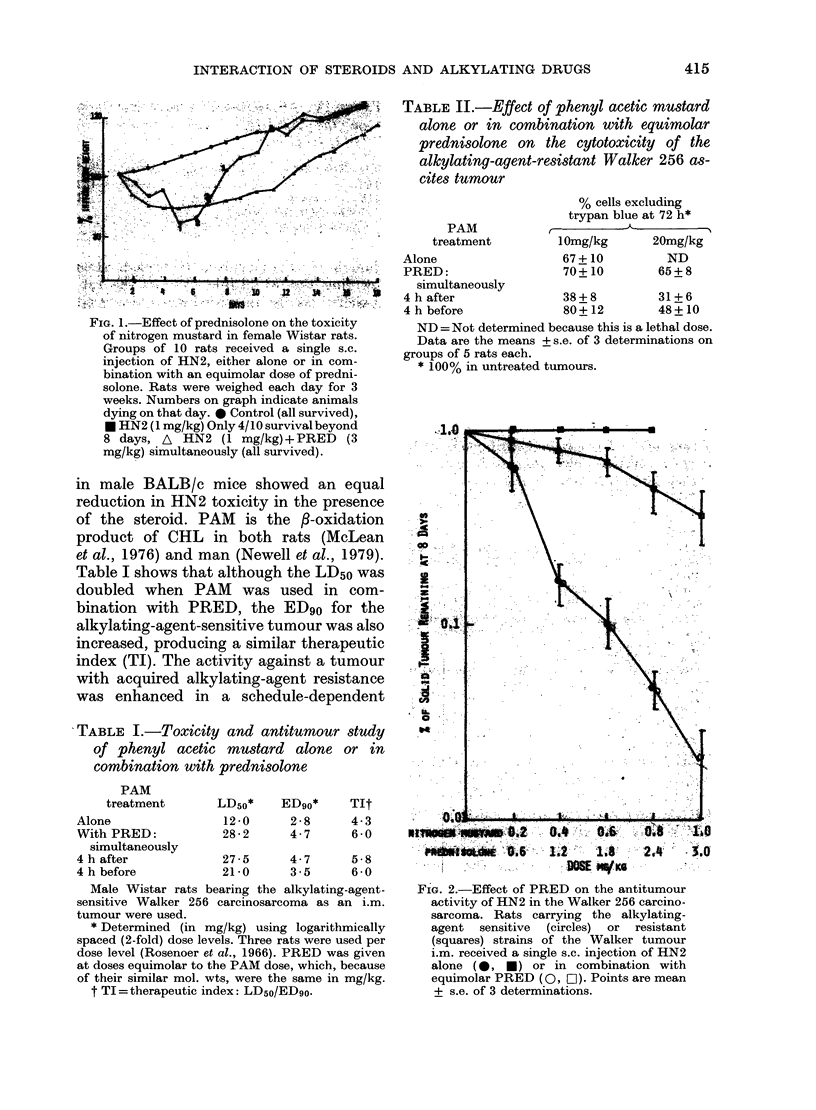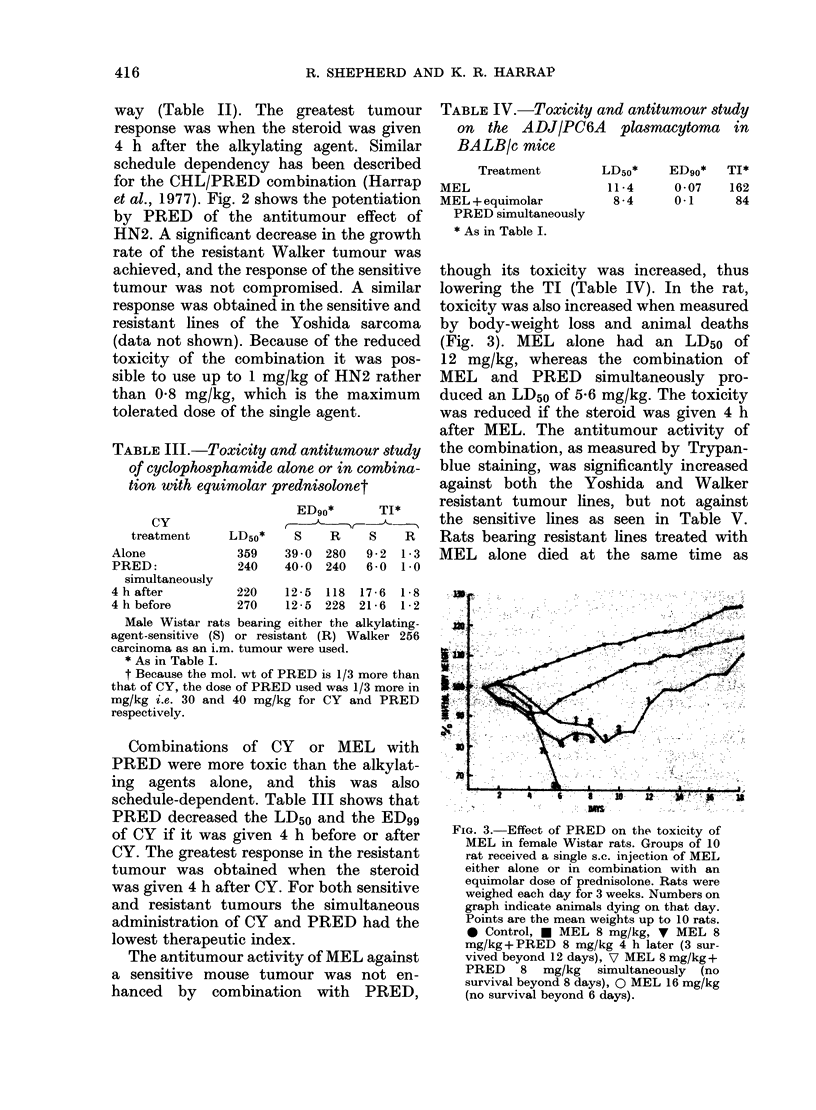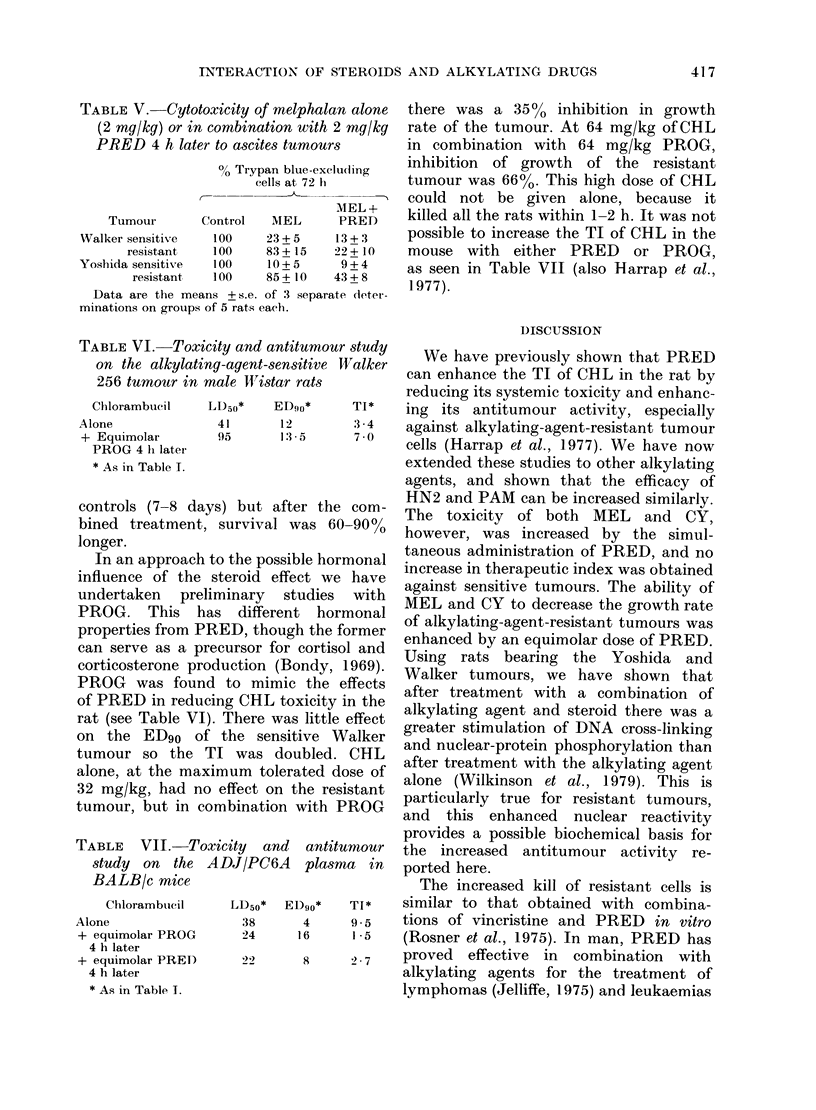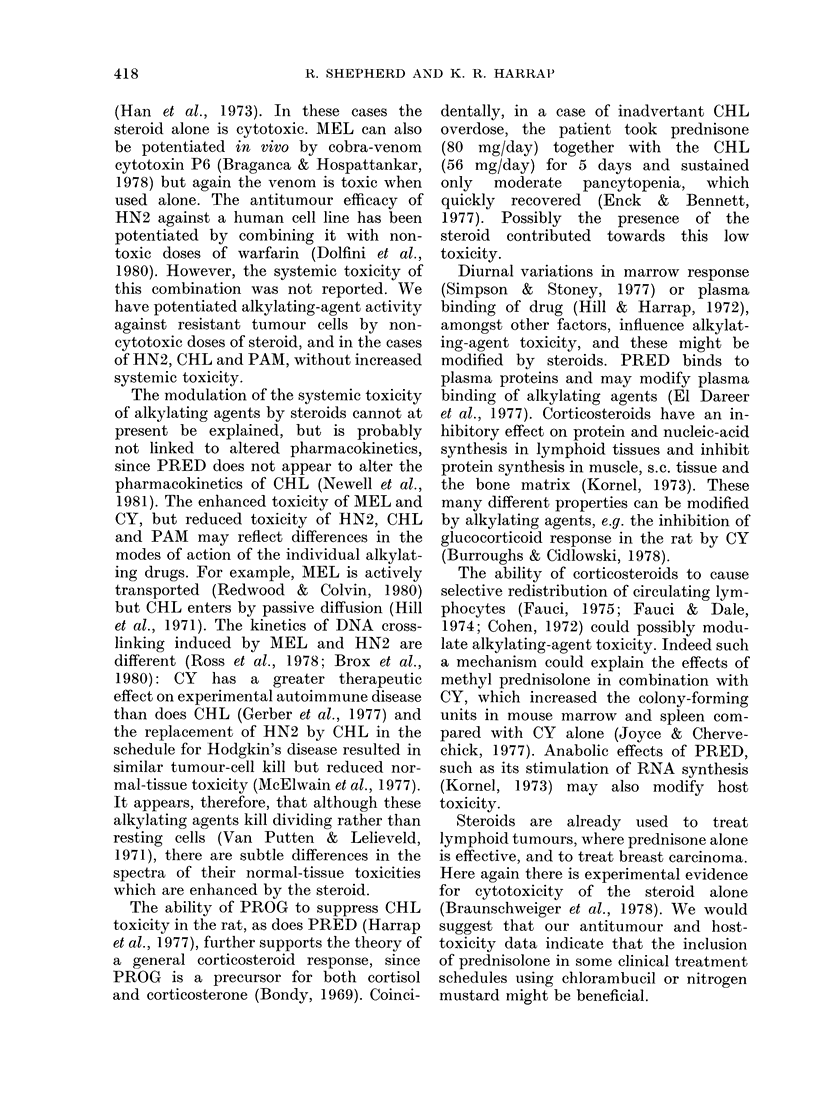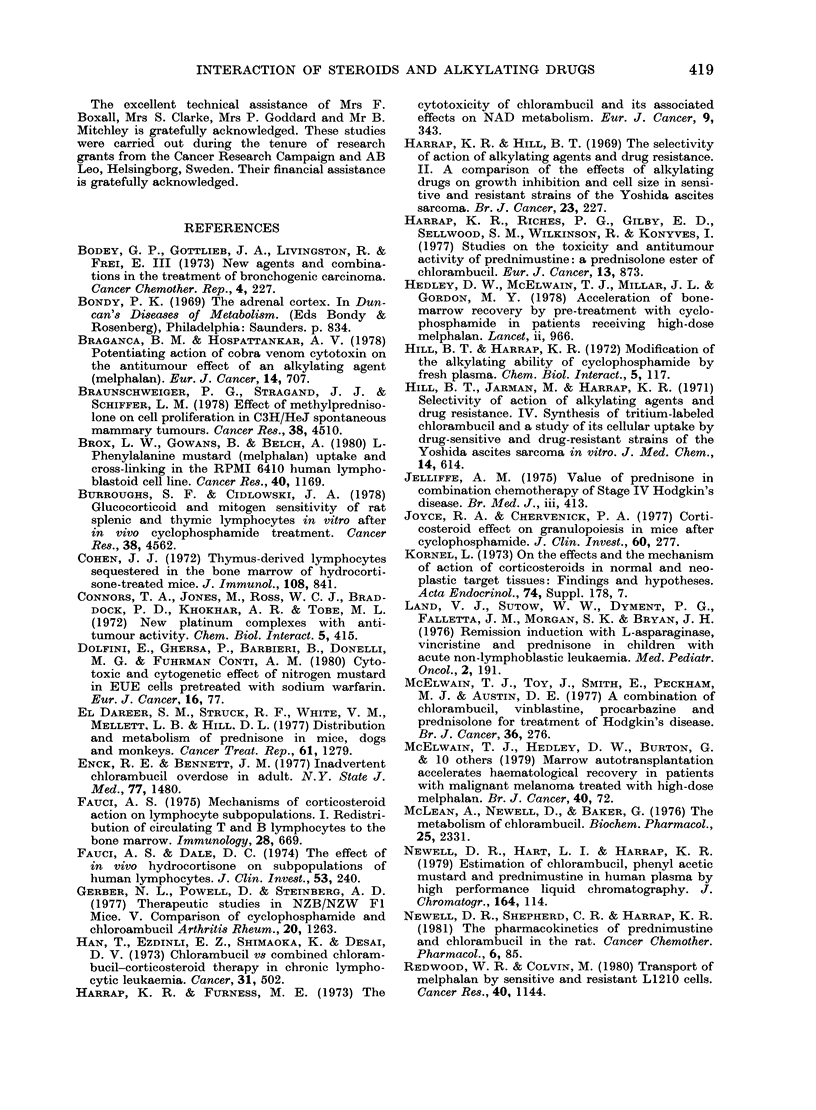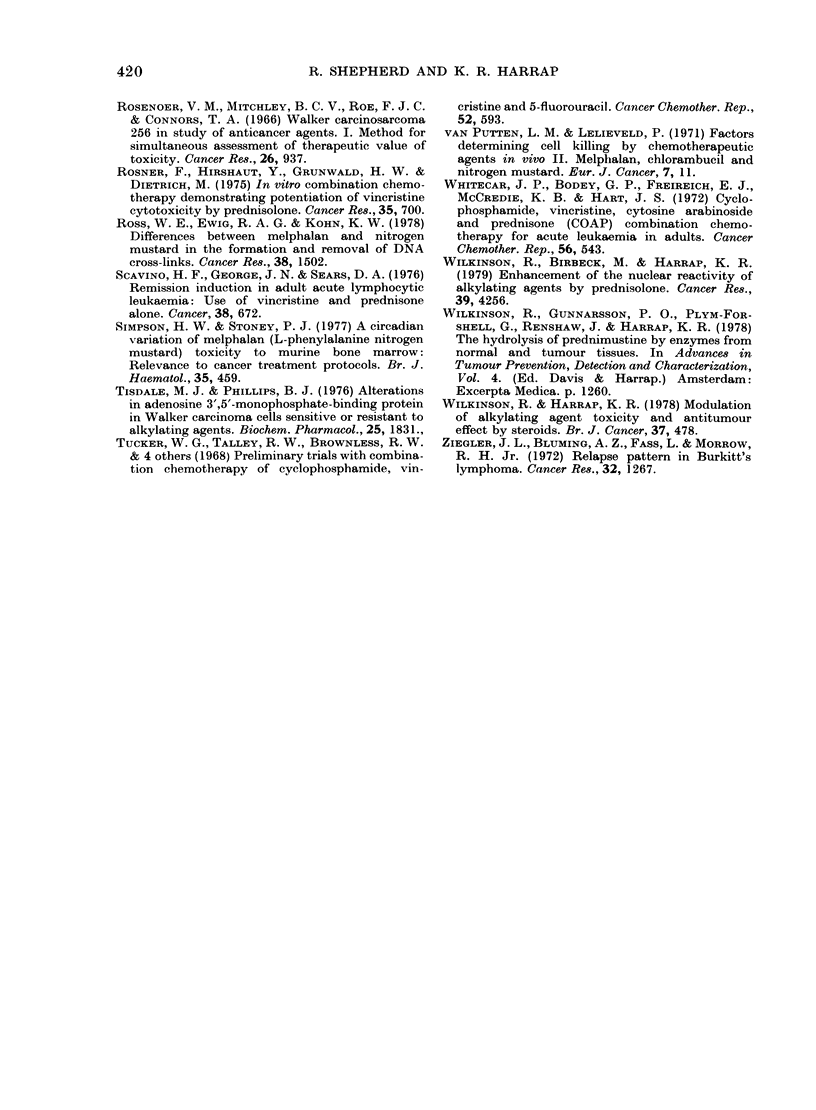# Modulation of the toxicity and antitumour activity of alkylating drugs by steroids.

**DOI:** 10.1038/bjc.1982.69

**Published:** 1982-03

**Authors:** R. Shepherd, K. R. Harrap

## Abstract

The steroids prednisolone and progesterone significantly altered the therapeutic indices of the alkylating agents, nitrogen mustard, melphalan, cyclophosphamide, phenyl acetic mustard and chlorambucil. For nitrogen mustard, chlorambucil and phenyl acetic mustard, prednisolone reduced host toxicity in the rat and enhanced the antitumour effectiveness against alkylating-agent-resistant strains of the Yoshida sarcoma and Walker carcinosarcoma. Progesterone also increased the therapeutic index of chlorambucil in the rat by decreasing its systemic toxicity. Two other alkylating agents, melphalan and cyclophosphamide, exhibited lower therapeutic indices in combination with prednisolone against alkylating-agent-sensitive tumours. This was due to the greater host toxicity of the combination than of the alkylating agent alone. In alkylating-agent-resistant tumours, however, a significant increase in growth delay was achieved if prednisolone was combined with the alkylating agent.


*Br. J. Cancer* (1982) **45**, 413

### MODULATION OF THE TOXICITY AND ANTITUMOUR ACTIVITY OF ALKYLATING DRUGS BY STEROIDS

R. SHEPHERD* AND K. R. HARRAP

*From the Department of Biochemical Pharmacology Institute of Cancer Research, Sutton, Surrey*

Received 29 February 1981 Accepted 10 November 1981

**Summary.—The steroids prednisolone and progesterone significantly altered the therapeutic indices of the alkylating agents, nitrogen mustard, melphalan, cyclophosphamide, phenyl acetic mustard and chlorambucil. For nitrogen mustard, chlorambucil and phenyl acetic mustard, prednisolone reduced host toxicity in the rat and enhanced the antitumour effectiveness against alkylating-agent-resistant strains of the Yoshida sarcoma and Walker carcinosarcoma. Progesterone also increased the therapeutic index of chlorambucil in the rat by decreasing its systemic toxicity.**

**Two other alkylating agents, melphalan and cyclophosphamide, exhibited lower therapeutic indices in combination with prednisolone against alkylating-agent-sensitive tumours. This was due to the greater host toxicity of the combination than of the alkylating agent alone. In alkylating-agent-resistant tumours, however, a significant increase in growth delay was achieved if prednisolone was combined with the alkylating agent.**

IN THE TREATMENT of malignant diseases, alkylating agents, intercalating antibiotics, antimetabolites and *Vinca* alkaloids are often used in combination (Ziegler *et al.*, 1972; Tucker *et al.*, 1968; Bodey *et al.*, 1973). However, the refractive nature of many tumours, and the heterogeneity of their drug sensitivity, has necessitated the use of increasingly high doses of drugs to achieve a tumour response. The margin of tolerance between tumour response and the systemic toxicity to the host has therefore narrowed. The use of pretreatment with cyclophosphamide (Hedley *et al.*, 1978) and marrow autotransplantation (McElwain *et al.*, 1979) have been used successfully in combination with high-dose melphalan, but there is a clear need for other approaches to widen the therapeutic gap.

We have previously shown that prednisolone is able to improve the therapeutic index of chlorambucil in an experimental tumour system (Harrap *et al.*, 1977). Host toxicity was reduced and tumour-cell kill maintained. Further, a tumour with acquired resistance which was refractory to chlorambucil alone responded to the combined treatment. Prednisolone is frequently included in clinical treatment schedules especially for tumours of the lympho-proliferative system, where tumoricidal effects could be expected from the corticosteroid alone (Scavino *et al.*, 1976; Land *et al.*, 1976; Whitecar *et al.*, 1972). In our experimental system, however, the tumour did not respond to prednisolone alone (Harrap *et al.*, 1977). In view of the extensive clinical use of alkylating drugs we thought it important to determine whether the enhanced therapeutic index in a non-steroid-responsive tissue could be extended to other alkylating agent/steroid combinations or whether it was a property peculiar to chlorambucil.

* To whom all correspondence should be addressed.

We now present data on the toxicity and antitumour activity of a number of alkylating agent/steroid combinations in 2 different experimental tumour systems *in vivo*. An abstract of preliminary results of this study has already been published (Wilkinson & Harrap, 1978).

### MATERIALS AND METHODS

Chlorambucil (4,'-(2-dichloroethyl)-amino)-phenyl butyrate, CHL) was a generous gift from the Wellcome Foundation, Beckenham, Kent. Melphalan (L,p-(2-dichloroethyl)-amino)-phenyl alanine, MEL) and phenyl acetic mustard, 4,p-(2-(dichloroethyl)-amino)-phenyl acetate, PAM) were synthesized in the Chester Beatty Research Institute (London). Nitrogen mustard (methyl-bis-chloroethyl amine, HN2) was purchased from The Boots Co. Ltd (Nottingham) and cyclophosphamide (2-(bis (2-chloroethyl)-amino)-2H-1,3,2-oxazaphosphorinane 2-oxide, CY) from WB Pharmaceuticals (Bracknell, Berks). Prednisolone (PRED) was purchased from Sigma (Kingston-upon-Thames) and progesterone (PROG) was obtained from Organon Laboratories Ltd (Morden, Surrey).

All other chemicals were purchased from either Hopkin and Williams (Chadwell Heath, Essex) or BDH (Poole, Dorset), AnalaR grades being used where available.

*Tumour lines.*—Wistar rats were used for all studies on the Yoshida sarcoma and Walker 256 carcinosarcoma. Two lines of Yoshida sarcoma ascites, one sensitive to alkylating agents and one with a 50-fold acquired resistance (Harrap & Furness, 1973), were routinely passaged weekly. Tumour cells were removed from the peritoneal cavity using a sterile 0·9% NaCl solution, and an aliquot counted with a Coulter electronic particle counter (Model ZF). Aliquots of tumour cells ($10^6$, $2 \times 10^6$ or $10^7$) were injected i.p. into recipient rats (Harrap & Hill, 1969).

The resistant strain of the Walker carcinosarcoma was developed *in vitro* by repeated CHL treatments (Tisdale & Phillips, 1976) and both sensitive and resistant Walker tumours were passaged as ascites tumours as described for the Yoshida tumour. The ADJ/PC6A plasmacytoma was maintained as a solid tumour passaged s.c. every 21 days by trochar as a $1 mm^3$ implant fragment (Connors *et al.*, 1972).

*Antitumour studies and drug treatments.*—For antitumour studies on the Yoshida sarcoma the cells in the peritoneal cavity were counted each day for 3 days after drug treatments, as previously described (Harrap & Hill, 1969). Drugs were administered s.c. in the back of the neck 3 days after tumour implant. Studies on the Walker tumour were performed in two ways. The tumour was injected i.p. as an ascites and cells counted for 3 days, as for the Yoshida tumour. In these cases rats received s.c. drug treatments in the back of the neck. Or the Walker tumour cells were injected i.m. in one hind leg, where a solid tumour developed. The untreated tumour grew to a weight of 9–10 g in 8 days. The weight of tumour in the drug-treated animals was compared with that of the untreated controls 8 days after tumour implant. Rats bearing the solid tumours were treated i.p. or s.c. one day after tumour implant (Rosenoer *et al.*, 1966). The ADJ/PC6A tumour was implanted s.c. in the flank, and drug treatments were given i.p. 24 days later. The weights of tumour in drug-treated and control mice were compared 10 days after drug treatment, when the untreated tumours weighed 8–9 g (Connors *et al.*, 1972). CHL and PRED were dissolved in dimethyl sulphoxide (DMSO), MEL in ethanolic HCl-phosphate, propylene glycol (Harrap & Hill, 1969) and CY and PROG in 0·9% NaCl. The steroids were always given in equimolar doses to those of the alkylating agent, because this combination had proved effective in the form of the steroid ester prednimustine, which is inactive until hydrolysed to its component molecules (Wilkinson *et al.*, 1978; Harrap *et al.*, 1977).

### RESULTS

In the rat, the systemic toxicity of either HN2 (Fig. 1) or PAM (Table I) was reduced if the alkylating agent was administered in combination with PRED. Fig. 1 details the systemic toxicity following a dose of 1 mg/kg of HN2, but no deaths occurred if this was accompanied by an equimolar (3 mg/kg) dose of PRED. The steroid was equally effective if given simultaneously with HN2 or 4 h later. Similar toxicity studies

FIG. 1.—Effect of prednisolone on the toxicity of nitrogen mustard in female Wistar rats. Groups of 10 rats received a single s.c. injection of HN2, either alone or in combination with an equimolar dose of prednisolone. Rats were weighed each day for 3 weeks. Numbers on graph indicate animals dying on that day. ● Control (all survived), ■ HN2 (1 mg/kg) Only 4/10 survival beyond 8 days, △ HN2 (1 mg/kg)+PRED (3 mg/kg) simultaneously (all survived).

in male BALB/c mice showed an equal reduction in HN2 toxicity in the presence of the steroid. PAM is the $\beta$-oxidation product of CHL in both rats (McLean *et al.*, 1976) and man (Newell *et al.*, 1979). Table I shows that although the $LD_{50}$ was doubled when PAM was used in combination with PRED, the $ED_{90}$ for the alkylating-agent-sensitive tumour was also increased, producing a similar therapeutic index (TI). The activity against a tumour with acquired alkylating-agent resistance was enhanced in a schedule-dependent

TABLE I.—*Toxicity and antitumour study of phenyl acetic mustard alone or in combination with prednisolone*

| PAM treatment | $LD_{50}$* | $ED_{90}$* | TI† |
|---|---|---|---|
| Alone | 12·0 | 2·8 | 4·3 |
| With PRED: simultaneously | 28·2 | 4·7 | 6·0 |
| 4 h after | 27·5 | 4·7 | 5·8 |
| 4 h before | 21·0 | 3·5 | 6·0 |

Male Wistar rats bearing the alkylating-agent-sensitive Walker 256 carcinosarcoma as an i.m. tumour were used.

* Determined (in mg/kg) using logarithmically spaced (2-fold) dose levels. Three rats were used per dose level (Rosenoer *et al.*, 1966). PRED was given at doses equimolar to the PAM dose, which, because of their similar mol. wts, were the same in mg/kg.

† TI = therapeutic index: $LD_{50}/ED_{90}$.

TABLE II.—*Effect of phenyl acetic mustard alone or in combination with equimolar prednisolone on the cytotoxicity of the alkylating-agent-resistant Walker* 256 *ascites tumour*

| PAM treatment | % cells excluding trypan blue at 72 h* | |
|---|---|---|
| | 10mg/kg | 20mg/kg |
| Alone | 67 ± 10 | ND |
| PRED: simultaneously | 70 ± 10 | 65 ± 8 |
| 4 h after | 38 ± 8 | 31 ± 6 |
| 4 h before | 80 ± 12 | 48 ± 10 |

ND = Not determined because this is a lethal dose. Data are the means ± s.e. of 3 determinations on groups of 5 rats each.

* 100% in untreated tumours.

FIG. 2.—Effect of PRED on the antitumour activity of HN2 in the Walker 256 carcinosarcoma. Rats carrying the alkylating-agent sensitive (circles) or resistant (squares) strains of the Walker tumour i.m. received a single s.c. injection of HN2 alone (●, ■) or in combination with equimolar PRED (○, □). Points are mean ± s.e. of 3 determinations.

way (Table II). The greatest tumour response was when the steroid was given 4 h after the alkylating agent. Similar schedule dependency has been described for the CHL/PRED combination (Harrap *et al.*, 1977). Fig. 2 shows the potentiation by PRED of the antitumour effect of HN2. A significant decrease in the growth rate of the resistant Walker tumour was achieved, and the response of the sensitive tumour was not compromised. A similar response was obtained in the sensitive and resistant lines of the Yoshida sarcoma (data not shown). Because of the reduced toxicity of the combination it was possible to use up to 1 mg/kg of HN2 rather than 0·8 mg/kg, which is the maximum tolerated dose of the single agent.

TABLE III.—*Toxicity and antitumour study of cyclophosphamide alone or in combination with equimolar prednisolone*†

| CY treatment | $LD_{50}$* | $ED_{90}$* S | $ED_{90}$* R | TI* S | TI* R |
|---|---|---|---|---|---|
| Alone | 359 | 39·0 | 280 | 9·2 | 1·3 |
| PRED: simultaneously | 240 | 40·0 | 240 | 6·0 | 1·0 |
| 4 h after | 220 | 12·5 | 118 | 17·6 | 1·8 |
| 4 h before | 270 | 12·5 | 228 | 21·6 | 1·2 |

Male Wistar rats bearing either the alkylating-agent-sensitive (S) or resistant (R) Walker 256 carcinoma as an i.m. tumour were used.
* As in Table I.
† Because the mol. wt of PRED is 1/3 more than that of CY, the dose of PRED used was 1/3 more in mg/kg *i.e.* 30 and 40 mg/kg for CY and PRED respectively.

Combinations of CY or MEL with PRED were more toxic than the alkylating agents alone, and this was also schedule-dependent. Table III shows that PRED decreased the $LD_{50}$ and the $ED_{99}$ of CY if it was given 4 h before or after CY. The greatest response in the resistant tumour was obtained when the steroid was given 4 h after CY. For both sensitive and resistant tumours the simultaneous administration of CY and PRED had the lowest therapeutic index.

The antitumour activity of MEL against a sensitive mouse tumour was not enhanced by combination with PRED,

TABLE IV.—*Toxicity and antitumour study on the ADJ/PC6A plasmacytoma in BALB/c mice*

| Treatment | $LD_{50}$* | $ED_{90}$* | TI* |
|---|---|---|---|
| MEL | 11·4 | 0·07 | 162 |
| MEL + equimolar PRED simultaneously | 8·4 | 0·1 | 84 |

* As in Table I.

though its toxicity was increased, thus lowering the TI (Table IV). In the rat, toxicity was also increased when measured by body-weight loss and animal deaths (Fig. 3). MEL alone had an $LD_{50}$ of 12 mg/kg, whereas the combination of MEL and PRED simultaneously produced an $LD_{50}$ of 5·6 mg/kg. The toxicity was reduced if the steroid was given 4 h after MEL. The antitumour activity of the combination, as measured by Trypan-blue staining, was significantly increased against both the Yoshida and Walker resistant tumour lines, but not against the sensitive lines as seen in Table V. Rats bearing resistant lines treated with MEL alone died at the same time as

FIG. 3.—Effect of PRED on the toxicity of MEL in female Wistar rats. Groups of 10 rat received a single s.c. injection of MEL either alone or in combination with an equimolar dose of prednisolone. Rats were weighed each day for 3 weeks. Numbers on graph indicate animals dying on that day. Points are the mean weights up to 10 rats. ● Control, ■ MEL 8 mg/kg, ▼ MEL 8 mg/kg + PRED 8 mg/kg 4 h later (3 survived beyond 12 days), ▽ MEL 8 mg/kg + PRED 8 mg/kg simultaneously (no survival beyond 8 days), ○ MEL 16 mg/kg (no survival beyond 6 days).

TABLE V.—*Cytotoxicity of melphalan alone (2 mg/kg) or in combination with 2 mg/kg PRED 4 h later to ascites tumours*

| Tumour | % Trypan blue-excluding cells at 72 h: Control | MEL | MEL+PRED |
|---|---|---|---|
| Walker sensitive | 100 | 23±5 | 13±3 |
| resistant | 100 | 83±15 | 22±10 |
| Yoshida sensitive | 100 | 10±5 | 9±4 |
| resistant | 100 | 85±10 | 43±8 |

Data are the means ±s.e. of 3 separate determinations on groups of 5 rats each.

TABLE VI.—*Toxicity and antitumour study on the alkylating-agent-sensitive Walker 256 tumour in male Wistar rats*

| Chlorambucil | $LD_{50}$* | $ED_{90}$* | TI* |
|---|---|---|---|
| Alone | 41 | 12 | 3·4 |
| + Equimolar PROG 4 h later | 95 | 13·5 | 7·0 |

* As in Table I.

controls (7–8 days) but after the combined treatment, survival was 60–90% longer.

In an approach to the possible hormonal influence of the steroid effect we have undertaken preliminary studies with PROG. This has different hormonal properties from PRED, though the former can serve as a precursor for cortisol and corticosterone production (Bondy, 1969). PROG was found to mimic the effects of PRED in reducing CHL toxicity in the rat (see Table VI). There was little effect on the $ED_{90}$ of the sensitive Walker tumour so the TI was doubled. CHL alone, at the maximum tolerated dose of 32 mg/kg, had no effect on the resistant tumour, but in combination with PROG there was a 35% inhibition in growth rate of the tumour. At 64 mg/kg of CHL in combination with 64 mg/kg PROG, inhibition of growth of the resistant tumour was 66%. This high dose of CHL could not be given alone, because it killed all the rats within 1–2 h. It was not possible to increase the TI of CHL in the mouse with either PRED or PROG, as seen in Table VII (also Harrap *et al.*, 1977).

TABLE VII.—*Toxicity and antitumour study on the ADJ/PC6A plasma in BALB/c mice*

| Chlorambucil | $LD_{50}$* | $ED_{90}$* | TI* |
|---|---|---|---|
| Alone | 38 | 4 | 9·5 |
| + equimolar PROG 4 h later | 24 | 16 | 1·5 |
| + equimolar PRED 4 h later | 22 | 8 | 2·7 |

* As in Table I.

### DISCUSSION

We have previously shown that PRED can enhance the TI of CHL in the rat by reducing its systemic toxicity and enhancing its antitumour activity, especially against alkylating-agent-resistant tumour cells (Harrap *et al.*, 1977). We have now extended these studies to other alkylating agents, and shown that the efficacy of HN2 and PAM can be increased similarly. The toxicity of both MEL and CY, however, was increased by the simultaneous administration of PRED, and no increase in therapeutic index was obtained against sensitive tumours. The ability of MEL and CY to decrease the growth rate of alkylating-agent-resistant tumours was enhanced by an equimolar dose of PRED. Using rats bearing the Yoshida and Walker tumours, we have shown that after treatment with a combination of alkylating agent and steroid there was a greater stimulation of DNA cross-linking and nuclear-protein phosphorylation than after treatment with the alkylating agent alone (Wilkinson *et al.*, 1979). This is particularly true for resistant tumours, and this enhanced nuclear reactivity provides a possible biochemical basis for the increased antitumour activity reported here.

The increased kill of resistant cells is similar to that obtained with combinations of vincristine and PRED *in vitro* (Rosner *et al.*, 1975). In man, PRED has proved effective in combination with alkylating agents for the treatment of lymphomas (Jelliffe, 1975) and leukaemias

(Han *et al.*, 1973). In these cases the steroid alone is cytotoxic. MEL can also be potentiated *in vivo* by cobra-venom cytotoxin P6 (Braganca & Hospattankar, 1978) but again the venom is toxic when used alone. The antitumour efficacy of HN2 against a human cell line has been potentiated by combining it with non-toxic doses of warfarin (Dolfini *et al.*, 1980). However, the systemic toxicity of this combination was not reported. We have potentiated alkylating-agent activity against resistant tumour cells by non-cytotoxic doses of steroid, and in the cases of HN2, CHL and PAM, without increased systemic toxicity.

The modulation of the systemic toxicity of alkylating agents by steroids cannot at present be explained, but is probably not linked to altered pharmacokinetics, since PRED does not appear to alter the pharmacokinetics of CHL (Newell *et al.*, 1981). The enhanced toxicity of MEL and CY, but reduced toxicity of HN2, CHL and PAM may reflect differences in the modes of action of the individual alkylating drugs. For example, MEL is actively transported (Redwood & Colvin, 1980) but CHL enters by passive diffusion (Hill *et al.*, 1971). The kinetics of DNA cross-linking induced by MEL and HN2 are different (Ross *et al.*, 1978; Brox *et al.*, 1980): CY has a greater therapeutic effect on experimental autoimmune disease than does CHL (Gerber *et al.*, 1977) and the replacement of HN2 by CHL in the schedule for Hodgkin's disease resulted in similar tumour-cell kill but reduced normal-tissue toxicity (McElwain *et al.*, 1977). It appears, therefore, that although these alkylating agents kill dividing rather than resting cells (Van Putten & Lelieveld, 1971), there are subtle differences in the spectra of their normal-tissue toxicities which are enhanced by the steroid.

The ability of PROG to suppress CHL toxicity in the rat, as does PRED (Harrap *et al.*, 1977), further supports the theory of a general corticosteroid response, since PROG is a precursor for both cortisol and corticosterone (Bondy, 1969). Coincidentally, in a case of inadvertant CHL overdose, the patient took prednisone (80 mg/day) together with the CHL (56 mg/day) for 5 days and sustained only moderate pancytopenia, which quickly recovered (Enck & Bennett, 1977). Possibly the presence of the steroid contributed towards this low toxicity.

Diurnal variations in marrow response (Simpson & Stoney, 1977) or plasma binding of drug (Hill & Harrap, 1972), amongst other factors, influence alkylating-agent toxicity, and these might be modified by steroids. PRED binds to plasma proteins and may modify plasma binding of alkylating agents (El Dareer *et al.*, 1977). Corticosteroids have an inhibitory effect on protein and nucleic-acid synthesis in lymphoid tissues and inhibit protein synthesis in muscle, s.c. tissue and the bone matrix (Kornel, 1973). These many different properties can be modified by alkylating agents, *e.g.* the inhibition of glucocorticoid response in the rat by CY (Burroughs & Cidlowski, 1978).

The ability of corticosteroids to cause selective redistribution of circulating lymphocytes (Fauci, 1975; Fauci & Dale, 1974; Cohen, 1972) could possibly modulate alkylating-agent toxicity. Indeed such a mechanism could explain the effects of methyl prednisolone in combination with CY, which increased the colony-forming units in mouse marrow and spleen compared with CY alone (Joyce & Chervechick, 1977). Anabolic effects of PRED, such as its stimulation of RNA synthesis (Kornel, 1973) may also modify host toxicity.

Steroids are already used to treat lymphoid tumours, where prednisone alone is effective, and to treat breast carcinoma. Here again there is experimental evidence for cytotoxicity of the steroid alone (Braunschweiger *et al.*, 1978). We would suggest that our antitumour and host-toxicity data indicate that the inclusion of prednisolone in some clinical treatment schedules using chlorambucil or nitrogen mustard might be beneficial.

The excellent technical assistance of Mrs F. Boxall, Mrs S. Clarke, Mrs P. Goddard and Mr B. Mitchley is gratefully acknowledged. These studies were carried out during the tenure of research grants from the Cancer Research Campaign and AB Leo, Helsingborg, Sweden. Their financial assistance is gratefully acknowledged.

### REFERENCES

BODEY, G. P., GOTTLIEB, J. A., LIVINGSTON, R. & FREI, E. III (1973) New agents and combinations in the treatment of bronchogenic carcinoma. *Cancer Chemother. Rep.*, **4**, 227.

BONDY, P. K. (1969) The adrenal cortex. In *Duncan's Diseases of Metabolism.* (Eds Bondy & Rosenberg), Philadelphia: Saunders. p. 834.

BRAGANCA, B. M. & HOSPATTANKAR, A. V. (1978) Potentiating action of cobra venom cytotoxin on the antitumour effect of an alkylating agent (melphalan). *Eur. J. Cancer*, **14**, 707.

BRAUNSCHWEIGER, P. G., STRAGAND, J. J. & SCHIFFER, L. M. (1978) Effect of methylprednisolone on cell proliferation in C3H/HeJ spontaneous mammary tumours. *Cancer Res.*, **38**, 4510.

BROX, L. W., GOWANS, B. & BELCH, A. (1980) L-Phenylalanine mustard (melphalan) uptake and cross-linking in the RPMI 6410 human lymphoblastoid cell line. *Cancer Res.*, **40**, 1169.

BURROUGHS, S. F. & CIDLOWSKI, J. A. (1978) Glucocorticoid and mitogen sensitivity of rat splenic and thymic lymphocytes *in vitro* after *in vivo* cyclophosphamide treatment. *Cancer Res.*, **38**, 4562.

COHEN, J. J. (1972) Thymus-derived lymphocytes sequestered in the bone marrow of hydrocortisone-treated mice. *J. Immunol.*, **108**, 841.

CONNORS, T. A., JONES, M., ROSS, W. C. J., BRADDOCK, P. D., KHOKHAR, A. R. & TOBE, M. L. (1972) New platinum complexes with anti-tumour activity. *Chem. Biol. Interact.* **5**, 415.

DOLFINI, E., GHERSA, P., BARBIERI, B., DONELLI, M. G. & FUHRMAN CONTI, A. M. (1980) Cytotoxic and cytogenetic effect of nitrogen mustard in EUE cells pretreated with sodium warfarin. *Eur. J. Cancer*, **16**, 77.

EL DAREER, S. M., STRUCK, R. F., WHITE, V. M., MELLETT, L. B. & HILL, D. L. (1977) Distribution and metabolism of prednisone in mice, dogs and monkeys. *Cancer Treat. Rep.*, **61**, 1279.

ENCK, R. E. & BENNETT, J. M. (1977) Inadvertent chlorambucil overdose in adult. *N.Y. State J. Med.*, **77**, 1480.

FAUCI, A. S. (1975) Mechanisms of corticosteroid action on lymphocyte subpopulations. I. Redistribution of circulating T and B lymphocytes to the bone marrow. *Immunology*, **28**, 669.

FAUCI, A. S. & DALE, D. C. (1974) The effect of *in vivo* hydrocortisone on subpopulations of human lymphocytes. *J. Clin. Invest.*, **53**, 240.

GERBER, N. L., POWELL, D. & STEINBERG, A. D. (1977) Therapeutic studies in NZB/NZW F1 Mice. V. Comparison of cyclophosphamide and chloroambucil *Arthritis Rheum.*, **20**, 1263.

HAN, T., EZDINLI, E. Z., SHIMAOKA, K. & DESAI, D. V. (1973) Chlorambucil *vs* combined chlorambucil–corticosteroid therapy in chronic lymphocytic leukaemia. *Cancer*, **31**, 502.

HARRAP, K. R. & FURNESS, M. E. (1973) The cytotoxicity of chlorambucil and its associated effects on NAD metabolism. *Eur. J. Cancer*, **9**, 343.

HARRAP, K. R. & HILL, B. T. (1969) The selectivity of action of alkylating agents and drug resistance. II. A comparison of the effects of alkylating drugs on growth inhibition and cell size in sensitive and resistant strains of the Yoshida ascites sarcoma. *Br. J. Cancer*, **23**, 227.

HARRAP, K. R., RICHES, P. G., GILBY, E. D., SELLWOOD, S. M., WILKINSON, R. & KONYVES, I. (1977) Studies on the toxicity and antitumour activity of prednimustine: a prednisolone ester of chlorambucil. *Eur. J. Cancer*, **13**, 873.

HEDLEY, D. W., MCELWAIN, T. J., MILLAR, J. L. & GORDON, M. Y. (1978) Acceleration of bone-marrow recovery by pre-treatment with cyclophosphamide in patients receiving high-dose melphalan. *Lancet*, ii, 966.

HILL, B. T. & HARRAP, K. R. (1972) Modification of the alkylating ability of cyclophosphamide by fresh plasma. *Chem. Biol. Interact.*, **5**, 117.

HILL, B. T., JARMAN, M. & HARRAP, K. R. (1971) Selectivity of action of alkylating agents and drug resistance. IV. Synthesis of tritium-labeled chlorambucil and a study of its cellular uptake by drug-sensitive and drug-resistant strains of the Yoshida ascites sarcoma *in vitro*. *J. Med. Chem.*, **14**, 614.

JELLIFFE, A. M. (1975) Value of prednisone in combination chemotherapy of Stage IV Hodgkin's disease. *Br. Med. J.*, iii, 413.

JOYCE, R. A. & CHERVENICK, P. A. (1977) Corticosteroid effect on granulopoiesis in mice after cyclophosphamide. *J. Clin. Invest.*, **60**, 277.

KORNEL, L. (1973) On the effects and the mechanism of action of corticosteroids in normal and neoplastic target tissues: Findings and hypotheses. *Acta Endocrinol.*, **74**, Suppl. 178, 7.

LAND, V. J., SUTOW, W. W., DYMENT, P. G., FALLETTA, J. M., MORGAN, S. K. & BRYAN, J. H. (1976) Remission induction with L-asparaginase, vincristine and prednisone in children with acute non-lymphoblastic leukaemia. *Med. Pediatr. Oncol.*, **2**, 191.

MCELWAIN, T. J., TOY, J., SMITH, E., PECKHAM, M. J. & AUSTIN, D. E. (1977) A combination of chlorambucil, vinblastine, procarbazine and prednisolone for treatment of Hodgkin's disease. *Br. J. Cancer*, **36**, 276.

MCELWAIN, T. J., HEDLEY, D. W., BURTON, G. & 10 others (1979) Marrow autotransplantation accelerates haematological recovery in patients with malignant melanoma treated with high-dose melphalan. *Br. J. Cancer*, **40**, 72.

MCLEAN, A., NEWELL, D., & BAKER, G. (1976) The metabolism of chlorambucil. *Biochem. Pharmacol.*, **25**, 2331.

NEWELL, D. R., HART, L. I. & HARRAP, K. R. (1979) Estimation of chlorambucil, phenyl acetic mustard and prednimustine in human plasma by high performance liquid chromatography. *J. Chromatogr.*, **164**, 114.

NEWELL, D. R., SHEPHERD, C. R. & HARRAP, K. R. (1981) The pharmacokinetics of prednimustine and chlorambucil in the rat. *Cancer Chemother. Pharmacol.*, **6**, 85.

REDWOOD, W. R. & COLVIN, M. (1980) Transport of melphalan by sensitive and resistant L1210 cells. *Cancer Res.*, **40**, 1144.

ROSENOER, V. M., MITCHLEY, B. C. V., ROE, F. J. C. & CONNORS, T. A. (1966) Walker carcinosarcoma 256 in study of anticancer agents. I. Method for simultaneous assessment of therapeutic value of toxicity. *Cancer Res.*, **26**, 937.

ROSNER, F., HIRSHAUT, Y., GRUNWALD, H. W. & DIETRICH, M. (1975) *In vitro* combination chemotherapy demonstrating potentiation of vincristine cytotoxicity by prednisolone. *Cancer Res.*, **35**, 700.

ROSS, W. E., EWIG, R. A. G. & KOHN, K. W. (1978) Differences between melphalan and nitrogen mustard in the formation and removal of DNA cross-links. *Cancer Res.*, **38**, 1502.

SCAVINO, H. F., GEORGE, J. N. & SEARS, D. A. (1976) Remission induction in adult acute lymphocytic leukaemia: Use of vincristine and prednisone alone. *Cancer*, **38**, 672.

SIMPSON, H. W. & STONEY, P. J. (1977) A circadian variation of melphalan (L-phenylalanine nitrogen mustard) toxicity to murine bone marrow: Relevance to cancer treatment protocols. *Br. J. Haematol.*, **35**, 459.

TISDALE, M. J. & PHILLIPS, B. J. (1976) Alterations in adenosine 3′,5′-monophosphate-binding protein in Walker carcinoma cells sensitive or resistant to alkylating agents. *Biochem. Pharmacol.*, **25**, 1831.,

TUCKER, W. G., TALLEY, R. W., BROWNLESS, R. W. & 4 others (1968) Preliminary trials with combination chemotherapy of cyclophosphamide, vincristine and 5-fluorouracil. *Cancer Chemother. Rep.*, **52**, 593.

VAN PUTTEN, L. M. & LELIEVELD, P. (1971) Factors determining cell killing by chemotherapeutic agents *in vivo* II. Melphalan, chlorambucil and nitrogen mustard. *Eur. J. Cancer*, **7**, 11.

WHITECAR, J. P., BODEY, G. P., FREIREICH, E. J., MCCREDIE, K. B. & HART, J. S. (1972) Cyclophosphamide, vincristine, cytosine arabinoside and prednisone (COAP) combination chemotherapy for acute leukaemia in adults. *Cancer Chemother. Rep.*, **56**, 543.

WILKINSON, R., BIRBECK, M. & HARRAP, K. R. (1979) Enhancement of the nuclear reactivity of alkylating agents by prednisolone. *Cancer Res.*, **39**, 4256.

WILKINSON, R., GUNNARSSON, P. O., PLYM-FORSHELL, G., RENSHAW, J. & HARRAP, K. R. (1978) The hydrolysis of prednimustine by enzymes from normal and tumour tissues. In *Advances in Tumour Prevention, Detection and Characterization, Vol.* 4. (Ed. Davis & Harrap.) Amsterdam: Excerpta Medica. p. 1260.

WILKINSON, R. & HARRAP, K. R. (1978) Modulation of alkylating agent toxicity and antitumour effect by steroids. *Br. J. Cancer*, **37**, 478.

ZIEGLER, J. L., BLUMING, A. Z., FASS, L. & MORROW, R. H. Jr. (1972) Relapse pattern in Burkitt's lymphoma. *Cancer Res.*, **32**, 1267.